# Assessing muscle quality as a key predictor to differentiate fallers from non-fallers in older adults

**DOI:** 10.1007/s41999-024-01020-y

**Published:** 2024-08-03

**Authors:** Emeline Michel, Raphael Zory, Olivier Guerin, Frederic Prate, Guillaume Sacco, Fréderic Chorin

**Affiliations:** 1 Department of Geriatric Medicine, Université Côte d’Azur, Centre Hospitalier Universitaire de Nice, Clinique Gériatrique de Soins Ambulatoires, 06003 Nice, France; 2 Université Côte d’Azur, LAMHESS, Nice, France; 3 Université Côte d’Azur, INSERM, CNRS, Nice, France; 4 Université Côte d’Azur, UPR 7276 CoBTek, Nice, France; 5 Institut Universitaire de France (IUF), Paris, France

**Keywords:** Muscle quality, Muscle power, Older adults, Fall, Aging

## Abstract

**Background:**

Falling is an important public health issue because of its prevalence and severe consequences. Evaluating muscle performance is important when assessing fall risk. The study aimed to identify factors [namely muscle capacity (strength, quality, and power) and spatio-temporal gait attributes] that best discriminate between fallers and non-fallers in older adults. The hypothesis is that muscle quality, defined as the ratio of muscle strength to muscle mass, is the best predictor of fall risk.

**Methods:**

184 patients were included, 81% (*n* = 150) were women and the mean age was 73.6 ± 6.83 years. We compared body composition, mean grip strength, spatio-temporal parameters, and muscle capacity of fallers and non-fallers. Muscle quality was calculated as the ratio of maximum strength to fat-free mass. Mean handgrip and power were also controlled by fat-free mass. We performed univariate analysis, logistic regression, and ROC curves.

**Results:**

The falling patients had lower muscle quality, muscle mass-controlled power, and mean weighted handgrip than the non-faller. Results showing that lower muscle quality increases fall risk (effect size = 0.891). Logistic regression confirmed muscle quality as a significant predictor (*p* < .001, OR = 0.82, CI [0.74; 0.89]). ROC curves demonstrated muscle quality as the most predictive factor of falling (AUC = 0.794).

**Conclusion:**

This retrospective study showed that muscle quality is the best predictor of fall risk, above spatial and temporal gait parameters. Our results underscore muscle quality as a clinically meaningful assessment and may be a useful complement to other assessments for fall prevention in the aging population.

## Key summary points

**Aim:**

The study aimed to identify factors, specifically muscle capacity (strength, quality, and power) and spatio-temporal gait attributes, that best discriminate between fallers and non-fallers in older adults.

**Findings:**

Falling patients exhibited lower muscle quality, muscle mass-controlled power, and mean weighted handgrip compared to non-fallers. Muscle quality was confirmed as a significant predictor of fall risk (*p* < .001, OR = 0.82, CI [0.74; 0.89]) and the most predictive factor (AUC = 0.794).

**Message:**

Muscle quality is the most effective predictor of fall risk and should be a key assessment component for fall prevention in the aging population.

## Introduction

Falls and their consequences (e.g., physical trauma and restriction of activity) are among the principal causes of morbidity in older adults [1]. Falling is a common geriatric syndrome due to its frequency and multifactorial causes [2]. Risk factors for falls are divided in two groups: (1) intrinsic risk factors linked to the person’s state of health, and (2) environmental risk factors linked to the characteristics of the place of fall. Intrinsic risk factors are considered to be the main causes of falls in older adults [2]. Aging of the neuromuscular system is an important intrinsic risk factor for falls among seniors [3]. The major components currently known to link aging and falls are a decline in muscle strength and in muscle mass [4]. Age-related loss of muscle function involves quantitative and qualitative changes in skeletal muscle structure, function [5], or both (sarcopenia). As individuals age, they undergo various physiological changes that could lead to sarcopenia and thus to an increased risk of falls [6].

Sarcopenia is a progressive and generalized skeletal muscle disorder associated with increased likelihood of adverse outcomes including falls [6]. In 2018, EWGSOP2 improved the definition of sarcopenia and uses low muscle strength as the primary endpoint of sarcopenia; thus, muscle strength is currently the most reliable measure of muscle function [6]. Specifically, a sarcopenia diagnosis is confirmed by the presence of low muscle quantity or quality. By definition, this loss of muscle strength is the result of two main factors: (1) a reduction in muscle mass; (2) a loss of muscle quality (Muscle Quality = Muscular Strength /Muscle Quantity, therefore Muscular Strength = Muscle Quality × Muscle Quantity).

Recent years have seen a significant increase in literature on the subject. While initial research suggested that the deterioration of strength, and consequently functional ability, was attributed to muscle mass reduction, a number of contemporary studies challenge this idea [7, 8]. They suggest instead that muscle quality is the primary determinant. The term “muscle quality” has been originally introduced to refer to the relationship between muscle strength and muscle volume [9, 10]. There is no universal consensus on assessment methods for routine clinical practice [6], but muscle quality can be defined as the ratio of muscle strength to appendicular skeletal muscle mass [6]. Previous studies have emphasized the importance of muscle quality over muscle strength or muscle mass alone when assessing muscle performance among older people [11, 12]. Abe et al. showed that the relationship between grip strength and muscle thickness was a significant predictor of physical performance [12]. A recent cross-sectional study has shown that muscle quality is negatively associated with dynamic balance, fear of falling, and history of recurrent falls in older women [13]. Gadelha et al. also showed that low muscle quality was associated with a higher risk of falls [10]. Finally, Goodpaster et al. showed that the loss of muscle strength is more rapid than the loss of muscle mass, suggesting a decline in muscle quality [14]. However, the above-mentioned studies only evaluated the muscle quality in relation to the risk of falling and did not compare other parameters identified as other fall predictor factors, such as muscle power, grip strength, or spatio-temporal gait parameters.

The aim of this study was to identify the factors (muscle functionality and spatio-temporal gait attributes) that best discriminate between fallers and non-fallers in older adults. The main hypothesis is that muscle quality, defined as the ratio of muscle strength to muscle mass, is the best predictor of fall risk. Indeed, a decrease in muscle quality may precede the loss of muscle mass, enabling an earlier assessment of muscle impairment and thus preventive management of muscle mass loss.

## Methods

### Design and setting

This study was a descriptive, retrospective, observational, and single-center transversal case-control study, carried out within the day hospital facility in the Nice University Hospital Center between 1 September 2019 and 13 March 2020. Ethics Committee issued a favorable opinion (reference number 15089).

### Sample size calculation

According to Gadelha et al., worse muscle quality was associated with a higher risk of falling in older women (OR = 3.56) [13]. For this case-control study, we calculated the number of participants needed by applying the Case-Control Chi-square method with Yates continuity correction. Using an Alpha risk of 5%, a Power (1-beta) of 0.9, and 3 controls per case, the number of patients required for this study was 180 [45 fallers (cases) and 135 non-fallers (controls)].

### Participants

Patients aged over 65 years old and able to walk without walking aids assistance were included in the study. Patients suffering from a neurological disease, not affiliated to a Social Insurance, or under legal protection, were not included. All participants signed an informed consent.

### Protocol

Each patient evaluated at the day hospital benefits from a standardized assessment with a precise collection of the various analyses they undergo. The screening was conducted on a voluntary basis following a comprehensive geriatric assessment. We systematically collected the falls history from each participant’s medical records as part of this assessment. A fall was defined as an unintentional landing on the ground. All participants, regardless of their fall history, underwent the same standardized sequence of assessment in the following order: an impedancemetry, a measurement of grip strength, a quantified gait analysis, and an isokinetic evaluation.

#### Impedancemetry

This measurement was performed with an impedance meter (Quadscan 4000, Bodystat Ltd., Isle of Man, British Isles). The weight in kilograms (kg) and height in meters (*m*) of each of the participants were collected. Then, we calculated the body mass index (BMI) in kilograms per square meter according to the Quetelet formula: ${\text{BMI}} \left( {{\text{kg/}}m^{2} } \right) = \frac{{{\text{weight}} \left( {{\text{kg}}} \right)}}{{\left[ {{\text{height}} (m^{2} )} \right]}}$. The impedance measurement allowed us to collect the lean body mass, the fat-free mass, and the body fat mass in kilograms (kg). The fat free mass was calculated according to the prediction equation by Sergi et al [15]

$${\text{FFM}} \left( {{\text{kg}}} \right) = - 3.964 + (0.227 \times \left( {{\text{Height}}^{2} /{\text{Resistance}}} \right) + \left( {0.095 \times {\text{weight}}} \right) + \left( {1.384 \times {\text{sex}}} \right) + \left( {0.064 \times {\text{Reactance}}} \right)$$

(height in cm; resistance at 50 kHz in ohms; weight in kg; sex (male = 1 and female = 0); reactance at 50 kHz in ohms). These values were expressed as a percentage of the total mass.

#### Grip strength

The handgrip strength was measured using a manual dynamometer (MicroFET handgrip). The patient was sitting on a chair with the feet firmly on the floor and the back supported by the chair. The shoulder was held in adduction (elbows to the body), with no extension or flexion, and in neutral rotation. The elbow was kept at 90° of flexion and in neutral pronosupination. The wrist was also maintained in a neutral position. The assistant held slightly the elbow and the base of the dynamometer to avoid any position change. Each grip force measurement consisted of three readings of each limb alternated with a resting position. The best of the three maximum strength tests (from each hand) was selected. Data collected measured mean grip strength in Newton (*N*) and mean weighted grip strength calculated according to the handgrip to fat-free mass ratio (N/kg).

#### Gait analysis

Patients underwent a gait test with Optogait^®^ (Optogait, Microgate, Bolzano, Italy), a markerless system that utilizes bars equipped with LEDs and sensors to measure the distance between steps. Spatio-temporal gait parameters during a gait cycle were recorded over a distance of 10 m at comfort and maximal velocity. Five successive measurements were recorded for each velocity and were averaged. The Optogait system captures gait parameters by detecting interruptions in light beams as a person walks between two parallel sensor bars. Each footfall that interrupts a beam is recorded, allowing for accurate measurement of walk parameters. Data collected were gait speed (meters per second), cadence (steps per minute), and step lengths in meters. Single stance duration and the oscillation phase were calculated as a percentage of the gait cycle, with data recorded at a frequency of 1000 Hz.

#### Isokinetic dynamometer

Isokinetic dynamometer measurements were carried out on the dominant lower limb using the Biodex System 4 dynamometer (Biodex Medical Systems, Inc, Shirley, NY) to specifically assess knee extension. Each patient was placed on the device with the lever arm adjusted to their height. Participants were seated in an adjustable chair with the axis of rotation of the dynamometer aligned with the center of the knee joint. (i.e., lateral femoral condyle). The knee and thigh were fixed to the seat and to the tip of the of lever arm of the dynamometer. To restrict body movements, participants were strapped to the chair using broad straps across the pelvis and upper body. Finally, the arms were kept crossed over the torso to avoid any movements. Any tests that did not meet the required performance conditions were excluded and were repeated after a rest period of 4 min. To familiarize participants with isokinetic knee extension movements of the lower limbs, and to perform warm-ups with specific movements, participants did several sub-maximal practice repetitions to get familiar with the isokinetic device before starting the evaluation. Participants were then asked to perform three maximal knee extension contractions at six predefined speeds (180, 150, 120, 90, 60, and 30°/s). Only the best result of the three trials was used for statistical analysis. All participants were verbally encouraged in a standard manner during each test and a 4 min recovery period was strictly observed between each set of maximal extensions contractions to ensure optimal performance and recovery.

Data were recorded and stored on a computer, and were sampled at 100 Hz using an electronic interface card (Biodex Medical Systems Inc., X2151, Shirley, NY, USA). The maximal torque was identified as the highest value reached during the movement at each constant speed. The maximum instantaneous power was the product of torque and speed. The linear moment–speed relationship was calculated from the maximum torque value obtained at each speed. The power (*P*)–velocity (*V*) relation was based on a second-order polynomial

$$P = a \cdot V^{2} + b \cdot V + c,$$

where a, b, and c are the regression coefficients of the polynomial. From this equation, the maximum power (*P*_MAX_), and the corresponding optimum speed (*V*_OPT_) were determined as follows:

$$V_{{{\text{OPT}}}} = - \frac{b}{2a}\;{\text{and}}\;P_{{{\text{MAX}}}} = - \frac{{b^{2} }}{2a} + c.$$

The theoretical maximum moment (*M*_MAX_) and the theoretical maximum velocity (*V*_MAX_) were obtained by extrapolating the linear relation where it meets, respectively, the abscissa axis at *M* = 0 and the ordinate axis at *V* = 0. Optimal muscle strength has been identified as the force at which the muscle can generate maximum torque at optimal speed. All these relationships and parameters were processed using a Matlab script (R2008b, The Mathworks, Natick, Massachusetts, USA).

Data collected were maximum muscular strength (Newton meter), optimal muscular strength (Newton), maximal power (Watt), maximum velocity (degrees per second), and optimal speed (degrees per second), specific to knee extension movements. The maximal power was normalized by fat-free mass (obtained using an impedance meter) according to the formula

$${\text{Muscle mass}} - {\text{controlled power}} \left( {{\text{Watt}}/{\text{kg}}} \right) = \frac{{{\text{maximal power}} \left( {{\text{Watt}}} \right)}}{{{\text{fat}} - {\text{free mass }}\left( {{\text{kg}}} \right)}}.$$

Muscle quality can be defined as the ratio of muscle strength to appendicular skeletal muscle mass [6]. Therefore, the muscular quality was calculated according to the formula

$${\text{Muscle quality}} = \frac{{{\text{maximum muscle strength}} \left( {\text{Newton meter}} \right)}}{{{\text{fat}} - {\text{free mass}} \left( {{\text{kg}}} \right)}}.$$

#### Statistical analysis

All statistical analyses were performed using XLSTAT^®^ (version 2021.1.1.1089). For comparative analyses, two different groups were defined: non-fallers (no falls) and fallers. Descriptive statistics were calculated for both groups. The categorical variables are reported as absolute frequencies. Quantitative variables are described by the number of participants, mean, and standard deviation. The Kolmogorov–Smirnov test was used to assess the normality of the data. For continuous variables, parametric analyses were performed using the Student’s *t* test. For the independent samples *t* test, the effect size was determined by calculating Cohen’s d, that is, the mean difference between our two groups divided by the pooled standard deviation. We consider Cohen’s d of 0.2 to be a “small” effect, 0.5 to be “medium”, and 0.8 to be “large. For all statistical analyses, the significance threshold was set at *p* < *0.05*.

Logistic regression was performed to understand and predict the effect of one or more explicative variables on a binary response variable (fall or not). The most common functions used to link probability *p* to the explanatory variables are the logistic function (we refer to the Logit model). The significant variables obtained with the univariate analysis and not correlated with each other were included in a binary logistic regression model (BMI, fat mass, walking speed, double stance, muscle quality, and muscle power). The response variable was a binary qualitative variable: the presence of a fall. The explanatory variables are quantitative variables. We also performed a receiver-operating characteristic (ROC) curve to determine if the value of a quantitative parameter was able to accurately discriminate fallers and non-fallers. ROC curves were constructed for the significant variables in univariate analysis. Area under the curve (AUC) was used to compare the different tests with each other.

## Results

184 patients were included, 81% (*n* = 150) were women, and the mean age was 73.6 ± 6.83 years.

### Univariate analysis (Table 1)

**Table 1:** Comparative analysis of data between fallers and non-fallers

	Total (*n* = 184)	Fallers (*n* = 46)	Non-fallers (*n* = 138)	Test result	Effect size	*p* value
Socio-demographic						
Age (years ± SD)^**£**^	73.64 ± 6.83	73.72 ± 7.49	73.62 ± 6.63	*t* = − 0.09; [− 2.40;2.20]	0.01	0.931
Gender (female. %)¥	150 (81.5)	41 (89.1)	109 (78.9)	χ^2^ = 2.36		0.125
Weight (kg ± SD)^£^*	64.58 ± 11.06	69.82 ± 10.66	62.84 ± 10.67	*t* = − 3.85; [− 10.57; − 3.40]	0.655	< 0.001*
BMI (kg/m^2^ ± SD)^£^*	24.53 ± 4.04	26.22 ± 3.77	23.97 ± 3.98	*t* = − 3.35; [− 3.56; − 0.92]	0.578	0.001*
Handgrip						
Mean handgrip (N ± SD)^£^	225.16 ± 80.96	210.73 ± 66.07	229.97 ± 85.02	*t* = 1.39; [− 7.89;46.36]	0.253	0.164
Mean weighted handgrip (N/kg ± SD)^£^*	39.89 ± 18.45	31.91 ± 18.21	42.56 ± 17.81	*t* = 3.49; [4.63;16.67]	0.591	0.001*
Impedance						
Fat mass (% ± SD)^£^*	36.78 ± 7.78	39.44 ± 5.55	35.90 ± 8.23	*t* = − 2.71; [− 6.11; − 0.96]	0.498	0.007*
Lean mass (% ± SD)^£^*	63.29 ± 8.08	61.07 ± 6.55	64.05 ± 8.42	*t* = 2.19; [0.29;5.67]	0.637	0.030*
Fat-free mass (% ± SD)^£^*	9.81 ± 3.38	10.71 ± 2.96	9.50 ± 3.45	*t* = − 2.12; [− 2.33; − 0.08]	0.374	0.035*
Spatio-temporal parameters						
Walking speed (m/s ± SD)^£^*	1.19 ± 0.22	1.14 ± 0.19	1.22 ± 0.22	*t* = 2.18; [0.01;0.15]	0.380	0.031*
Cadence (step/min ± SD)^£^	113.82 ± 10.76	111.94 ± 9.81	114.45 ± 11.02	*t* = 1.38; [− 1.09;6.12]	0.241	0.171
Step length (cm ± SD)^£^	62.75 ± 8.44	60.68 ± 7.27	63.44 ± 8.72	*t* = 1.94; [− 0.05;5.58]	0.345	0.054
Double stance (% ± SD)^£^*	24.77 ± 4.88	26.55 ± 5.28	24.18 ± 4.61	*t* = − 2.90; [− 3.98;− 0.76]	0.466	0.004*
Simple support (% ± SD)^£^*	37.58 ± 2.49	36.69 ± 2.62	37.88 ± 2.38	*t* = 2.85; [0.36;2.00]	0.473	0.005*
Oscillating (% ± SD)^£^*	37.51 ± 2.49	36.60 ± 2.74	37.81 ± 2.33	*t* = 2.92; [0.39;2.03]	0.477	0.004*
Isokinetic dynamometer						
Muscle strength (Nm ± SD)^£^*	117.56 ± 38.14	107.42 ± 24.78	120.94 ± 41.17	*t* = 2.10; [0.83;26.22]	0.398	0.037*
Muscle quality (Nm/kg ± SD)^£^*	20.29 ± 7.17	15.84 ± 6.54	21.78 ± 6.77	*t* = 5.19; [3.68;8.19]	0.891	< 0.0001*
Muscle power (W ± SD)^£^*	165.99 ± 55.40	150.71 ± 44.98	171.09 ± 57.71	*t* = 2.18; [1.96;38.80]	0.394	0.030*
Muscle mass-controlled power (W/kg ± SD)^£^*	29.02 ± 11.87	22.04 ± 9.36	31.35 ± 11.73	*t* = 4.88; [5.55;13.06]	0.877	< 0.0001*
Optimal strength (Nm ± SD)^£^*	53.26 ± 18.08	46.87 ± 15.75	55.39 ± 18.35	*t* = 2.82; [2.56;14.48]	0.498	0.005*
Maximal speed (°/sec)^£^	322.69 ± 62.09	316.25 ± 71.67	324.84 ± 58.69	*t* = 0.81; [− 12.29;29.47]	0.131	0.418
Optimal speed (°/sec)^£^	173.84 ± 31.94	174.06 ± 43.37	173.76 ± 27.29	*t* = − 0.05; [− 11.06;10,46]	0.009	0.957

*£* quantitative data were compared using the Student t test and were expressed with Student t value and confidence interval at 95% [XX; YY], *¥* qualitative data were compared using Chi^2^ test and were expressed with Chi^2^ value

*SD* standard deviation, *kg* kilograms, *m* meter, *s* second, *cm* centimeter, *N* Newton, *Nm* Newton meter, *W* Watt

*Significant result* p* < *0.05*

The weight (*p* < 0.001), BMI (*p* = 0.001), fat-free mass (*p* = 0.035), and fat mass (*p* = 0.007) were significantly higher among fallers, with medium-effect size for weight and BMI and small effect size for impedance meter results. Lean mass and walking speed were lower among fallers (respectively, *p* = 0.030 and *p* = 0.031). Analysis of spatio-temporal gait parameters revealed a longer double stance time (*p* = 0.004), and a shorter simple stance (*p* = 0.005) and oscillating phase (*p* = 0.004) among fallers but a small effect size. The weighted mean grip strength was significantly lower among fallers (*p* < 0.001; effect size = 0.591). Finally, the results from the isokinetic dynamometer showed significantly lower maximum muscular strength (*p* = 0.037), optimal strength (*p* = 0.005), and muscle power (*p* = 0.003) among fallers, with small effect size. The muscle quality (*p* < 0.001) and muscle mass-controlled power (*p* < 0.001) were also lower, with large effect size.

### ROC curves (Fig. 1)

**Fig. 1:**
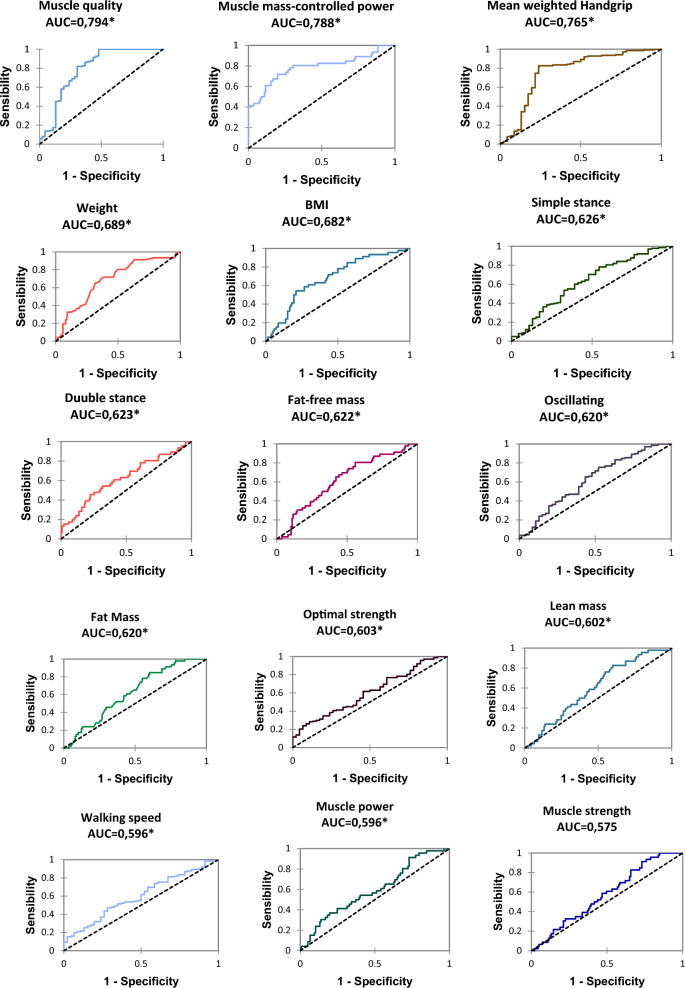
ROC curves. *BMI* body mass index, *significant result *p* < *0.05 AUC* area under the curve

The variable muscle quality has the highest area under the curve (0.794), with a threshold value of 13.35 Newton/kg. The comparison test was significant (*p* < 0.0001). Muscle mass-controlled power has a calculated AUC 0.788 with a threshold value of 23.41 Newton meter/kg. The comparison test was significant (*p* < 0.0001). The threshold value of the mean weighted grip force was 29.11 Newton meter/kg. The area under the curve was 0.765. The comparison test was significant (*p* < 0.0001). Finally, the following variables had a significant comparison test: weight (AUC = 0.689), BMI (AUC = 0.682), simple stance (AUC = 0.626), double stance (AUC = 0.623), fat-free mass (AUC = 0.622), oscillating phase (AUC = 0.620), fat mass (AUC = 0.620), optimum strength (AUC = 0.603), lean mass (AUC = 0.602), walking speed (AUC = 0.596), and muscle power (AUC = 0.596). All ROC curves are shown in Fig. 1.

### Logistic regression (Table 2 and Fig. 2)

**Table 2:** Logistic regression

Variable	Value	*p* value	OR	CI
BMI	0.072	0.381	1.074	[0.915;1.261]
Fat mass	0.021	0.638	1.021	[0.936;1.114]
Walking speed	− 0.851	0.505	0.427	[0.035;5.217]
Double stance	0.005	0.936	1.005	[0.894;1.130]
Muscle quality*	− 0.204	< 0.0001*	0.816	[0.741;0.897]
Muscle power*	− 0.013	0.038*	0.987	[0.976;0.999]

*OR* odds ratio, *CI* confidence interval, *BMI* body mass index,

^*^Significant result* p* < *0.05*

**Fig. 2:**
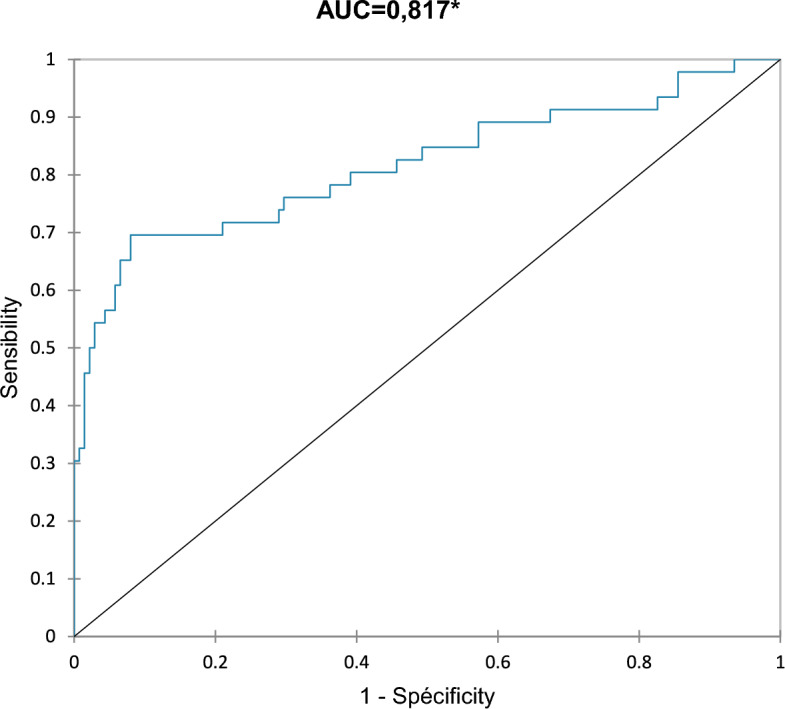
ROC curves: logistic regression

Results of the logistic regression were significant (*p* < 0.0001). The ROC curve established from the logistic regression model is presented in Fig. 2 (AUC = 0.817). Muscle quality has a negative impact on the risk of falling (− 0.204) (*p* < 0.0001*, OR = 0,82, CI [0.74; 0.89]). Muscle power also has a negative impact (− 0.013) (*p* = 0.038*, OR = 0.98, CI [0.98; 0.99]). The other variables were not significant (BMI, fat mass, walking speed, and double stance). The equation of the model was

$${\text{Pr}}\left( {{\text{Fall}}} \right) = \frac{1}{{({1 } + {\text{ exp}}( - \left( {{2}.{9} + 0,{1} \times {\text{BMI}} + 0.{1} \times {\text{fat mass}} - 0.{8} \times {\text{walking speed}} + 0.0{1} \times {\text{Double stance}} - 0.{2} \times {\text{quality}} - 0.0{1} \times {\text{power}}} \right)}}.$$

## Discussion

The aim of this study was to identify the parameters (muscle performance or spatio-temporal walking parameters) that best discriminate fallers and non-fallers in older adults. As hypothesized, muscle quality appears to be a determining factor in the risk of falling, with our results showing that lower muscle quality is associated with a higher risk of falling (effect size = 0.891). Muscle quality is also significant with logistic regression (*p* < 0.001, OR = 0.82, CI [0.74; 0.89]). On the other hand, muscle power showed significance in univariate analysis (*p* = 0.030), albeit with a smaller effect size. It also exhibited significance in logistic regression (*p* < 0.038, OR = 0.98, CI [0.97; 0.99]), and demonstrated significance in ROC curves (AUC = 0.623).

Muscle quality is the most predictive risk factor for falls, as demonstrated by various statistical analyses. These results confirm those of the literature showing that muscle quality is an important factor on physical function in frail, obese, older adults [16, 17] and that muscle quality is strongly related to an increased risk of falling in older adults [18] and older women (68 ± 6.2 years) [10]. This study is the first to compare muscle quality with other risk factors such as muscle power, grip strength, or spatio-temporal gait parameters in elderly people of both sexes. This is noteworthy, because, although muscular strength has been associated with better performance on functional tasks [19], an assessment of muscle quality, rather than a muscle strength evaluation, may be more appropriate. Additionally, we found differences in patients’ weights, leading to variations in impedance measurements. Fallers often had a higher BMI, fat mass, and lean mass. While a higher body mass index can be associated with thicker muscles [13], it is evident that muscle strength is a more crucial health indicator than muscle size in older individuals [20, 21]. Notably, just having more muscle does not mean that it is stronger, especially since strength and size do not always correlate linearly [14]. The significant decline in strength, even without a matching decrease in muscle mass, highlights the importance of muscle quality [17]. Also, individuals who fall more frequently tend to have a higher BMI, pointing to the issue of sarcopenic obesity [22]. This mix of weaker muscles and increased weight can lead to further functional decline [23].

In older individuals, muscle quality deteriorate due to several factors, including fat infiltration in skeletal muscles and the decrease in Type II fibers [24]. Aging also leads to a reduction in motor units [25], incomplete re-innervation [26], and reorganization of the remaining motoneurons [27], which affects muscle functionality [28]. Finally, a deficit in motor unit activation is commonly observed [29].

Thus, muscular quality, which encompasses various aspects of muscle function and performance, including strength, muscle composition, functional performance, and other neuromuscular factors, should be considered a more relevant indicator of fall risk than parameters, such as muscle strength or power. Indeed, muscular quality is defined as the ability of a muscle to generate force per unit of muscle mass, and it is influenced not only by a muscle’s strength or power but also by other factors, such as muscle composition, neuromuscular coordination, and the presence of fibrosis or fat infiltration. By integrating these elements, muscular quality provides a more comprehensive and nuanced view of an older individual’s motor capabilities. Several studies have suggested that superior muscular quality may compensate for lower muscle quantity [30, 31], meaning that muscles of “good quality” can effectively and precisely respond to functional demands, which are essential for maintaining balance and preventing falls. Although muscular quality is a key factor, other aspects, particularly muscle power, should not be overlooked when assessing fall risk.

Muscle power is also a relevant parameter in the risk of falling (effect size = 0.394) and significant in logistic regression (*p* = 0.038) and muscle mass-controlled power was significantly lower among fallers (*p* < 0.001) with a large effect size (0.877). These results underscore the importance of assessing overall muscle function. Power is characterized by the ability to generate energy in the shortest possible time. Power is also linked to strength (*F*) and velocity (*v*) through the relationship *p* = *F* x *v*. This relationship demonstrates that power decreases with both strength and velocity. Velocity is essential in situations where a quick reaction is needed to prevent a fall. Therefore, higher muscle power enables a swift and efficient response. Previous studies have already established a connection between muscle power and falls [32–34]. Power, when considered relative to strength, indicates impaired ability to contract rapidly [35]. Compared to strength or muscle quality, power suggests reduced contraction speed. With aging, there is a decline in the muscle’s ability to contract rapidly, which affects power. This decline is often attributed to various physiological changes, particularly the loss of fast-twitch muscle fibers and alterations in neuromuscular signaling pathways. Thus, muscle power is critical for assessing the risk of falls in older individuals, because it is closely related to muscle contraction speed and responsiveness.

Finally, the mean weighted grip strength was associated with a risk of falling, whereas the simple grip strength was not. Indeed, this parameter is significant in the univariate analysis (*p* = 0.001). Furthermore, ROC curves confirmed these results (AUC = 0.765). It is known that weak grip strength is a strong predictor of an increase in the duration of hospitalization, functional limitations, poor quality of life, and death [36, 37]. Of note, grip strength and lower extremity muscle strength have shown moderate-to-strong correlations in older adults [38]. However, studies suggest that grip strength should be used with caution to assess overall strength [39]. Finally, Ostolin et al. showed that the evolution of grip strength over time does not seem to predict the evolution of lower limb strength involved in the risk of falling [30]. Our findings are in line with these and also highlight the importance of muscle quality rather than muscle quantity.

Finally, there are significant differences in most of the spatio-temporal gait parameters between the two groups. In the non-faller group, walking speed was significantly higher (*p* = 0.031*, effect size = 0.380), the single stance time was longer (*p* = 0.005, effect size 0.473) and double stance was shorter (*p* = 0.004, effect size = 0.466). These are also variables obtained from ROC curves (DS: AUC = 0.623, single stance: AUC = 0.626, and oscillating: AUC = 0.620). This is consistent with the literature. Many studies have already shown the relationship between gait disorders and the risk of falling in older people, supporting the idea that decrease in gait speed and modification of gait parameters are strong predictors of falls [40, 41]. Thus, more than gait speed, it is the gait quality and single and double stance durations that is important. Variability in stride length and double stance duration are important predictors of gait among older adults [42]. However, gait parameters obtained with the Optogait are based on a number of limited and repeated stages and do not take into account other important variables such as joint angle [43]. Moreover, the walking speed and the time of double support did not appear significant in the logistic regression model. It is important to note that the previously cited studies did not compare all the parameters that we evaluated. Thus, more than walking speed, it is muscle function, and particularly muscle quality, that seems to be the most discriminating factor.

### Limits and biases

There could be several sources of bias in our study. Study was retrospective and a prospective study is necessary to establish temporal relationships between muscle quality and falls, as we did not have information on the implementation of preventive measures after previous falls. As with any observational study, it is impossible to assess risk factors and events preceding falls. While there is a significant link between muscle function and the risk of falling (with a clinical impact, in terms of prevention through adapted physical activity programs), no causal link between the actual observed fall parameters and their prior occurrence at the first fall can be established. Furthermore, retrospective fall history over 1 year is prone to both over- and underestimation. However, since the objective is to compare fallers and non-fallers, this does not impact the interpretation of the results.

Finally, the appropriate definition of muscle quality is a subject of ongoing debate. In our current study, muscle quality was defined as muscle strength expressed in relation to fat-free mass. Thus, a minor variation in fat-free mass results in a large variation in quality. This implies caution is needed in the interpretation of the results. Moreover fat-free muscle mass was obtained using bioelectrical impedance analysis. This method estimates muscle mass based on the body’s resistance to the flow of an electric current, which can sometimes lead to approximations. In future studies, it would be interesting to use computed tomography, magnetic resonance imaging, or dual-energy X-ray absorptiometry.

## Conclusions

In conclusion, this retrospective, observational study of older adults showed that muscle quality is the best predictor of fall risk, more than spatial and temporal gait parameters. Our results confirm that muscle quality is a clinically meaningful assessment and may be a useful complement to other assessments for fall prevention in the aging population. Further studies are needed to establish whether an increase in muscle quality could improve gait parameters and decrease fall risk. These results would be useful in recommending an appropriate physical activity program for fall prevention.
